# Research on a Joint Extraction Method of Track Circuit Entities and Relations Integrating Global Pointer and Tensor Learning

**DOI:** 10.3390/s24227128

**Published:** 2024-11-06

**Authors:** Yanrui Chen, Guangwu Chen, Peng Li

**Affiliations:** 1 School of Automation and Electrical Engineering, Lanzhou Jiaotong University, Lanzhou 730070, China; 11220430@stu.lzjtu.edu.cn (Y.C.); penglee_work@163.com (P.L.); 2 Key Laboratory of Plateau Traffic Information Engineering and Control of Gansu Province, Lanzhou Jiaotong University, Lanzhou 730070, China

**Keywords:** joint entity and relation extraction, track circuit, tucker decomposition, tensor learning, efficient global pointer

## Abstract

To address the issue of efficiently reusing the massive amount of unstructured knowledge generated during the handling of track circuit equipment faults and to automate the construction of knowledge graphs in the railway maintenance domain, it is crucial to leverage knowledge extraction techniques to efficiently extract relational triplets from fault maintenance text data. Given the current lag in joint extraction technology within the railway domain and the inefficiency in resource utilization, this paper proposes a joint extraction model for track circuit entities and relations, integrating Global Pointer and tensor learning. Taking into account the associative characteristics of semantic relations, the nesting of domain-specific terms in the railway sector, and semantic diversity, this research views the relation extraction task as a tensor learning process and the entity recognition task as a span-based Global Pointer search process. First, a multi-layer dilate gated convolutional neural network with residual connections is used to extract key features and fuse the weighted information from the 12 different semantic layers of the RoBERTa-wwm-ext model, fully exploiting the performance of each encoding layer. Next, the Tucker decomposition method is utilized to capture the semantic correlations between relations, and an Efficient Global Pointer is employed to globally predict the start and end positions of subject and object entities, incorporating relative position information through rotary position embedding (RoPE). Finally, comparative experiments with existing mainstream joint extraction models were conducted, and the proposed model’s excellent performance was validated on the English public datasets NYT and WebNLG, the Chinese public dataset DuIE, and a private track circuit dataset. The *F*1 scores on the NYT, WebNLG, and DuIE public datasets reached 92.1%, 92.7%, and 78.2%, respectively.

## 1. Introduction

As an essential component of the railway signaling system, the operational status of track circuit equipment is closely related to the safety and efficiency of railway traffic organization. The proper functioning of track circuits directly impacts train occupancy detection in sections and the transmission of traffic information. However, the high cost of track circuit fault maintenance, combined with frequent failures, makes it a critical focus of railway maintenance and protection efforts [1,2,3,4]. During the routine maintenance and inspections of track circuits, fault maintenance data are mostly stored in text form, containing information such as equipment status and fault-handling solutions. These data hold a wealth of knowledge related to track circuits, but due to the unstructured nature of the data, they are difficult to analyze and mine directly using computers, resulting in inefficiencies in reusing such data. Therefore, it is urgent to employ joint extraction techniques to automatically extract relevant entities and relationships from track circuit maintenance text data, transforming unstructured text into structured triplet knowledge that is easier to analyze and mine. This transformation will provide a knowledge base for constructing a knowledge graph in the railway maintenance domain, enabling the analysis of fault-related information, the identification of potential fault risks, and offering intelligent decision support for on-site track circuit maintenance [5].

**Figure 1:**
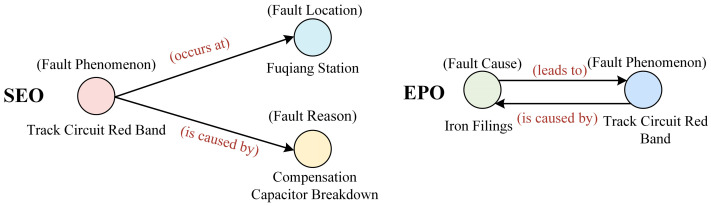
Example of overlapping relations.

Relation triplet extraction can be divided into two sub-tasks: entity recognition and relation extraction. Traditional pipeline extraction methods do not fully consider the feature correlations between these two sub-tasks. The relation extraction sub-task depends on the accuracy of the preceding entity recognition sub-task, which leads to the propagation and accumulation of errors [6,7,8]. To mitigate the shortcomings of pipeline extraction methods, researchers have proposed joint entity and relation extraction approaches, which are mainly divided into two types: parameter sharing-based joint extraction models and joint decoding-based models. However, the existing models still have several limitations: (1) In practical engineering applications, entities often have multiple relationships, resulting in overlapping and intersecting triplet information. The existing models cannot adaptively identify overlapping triplet information in sentences. For example, as shown in Figure 1, the overlapping relationship problem can be categorized into single entity overlap (SEO) and entity pair overlap (EPO). Triplet 1 (“track circuit red band”, “occurs at”, “Fuqiang Station”) and Triplet 2 (“track circuit red band”, “is caused by”, “compensation capacitor breakdown”) share the subject entity “track circuit red band”, indicating the presence of single entity overlap. Triplet 3 (“iron filings”, “leads to”, “track circuit red band”) and Triplet 4 (“track circuit red band”, “is caused by”, “iron filings”) exhibit entity pair overlap. (2) In recent years, many researchers have proposed various solutions to address these entity overlap scenarios. Some multi-stage learning methods have been developed to achieve parameter sharing and task interaction to a certain extent. However, these methods suffer from exposure bias, have a high time complexity, and exhibit a low generalization capability. (3) The existing models lack a sufficient exploration of the semantic relationships between multiple relations in the text. There may be some common patterns or associative characteristics between different types of relations, which could help improve the effectiveness of relation extraction. For instance, certain types of relationships exhibit semantic similarities, such as symmetry or inverse relations. An example of this is the triplets (“faulty circuit breaker”, “leads to”, “track circuit red band”) and (“track circuit red band”, “is caused by”, “faulty circuit breaker”), where the relation types “leads to” and “is caused by” have semantic associative characteristics.

To provide intelligent decision-making support for on-site maintenance personnel, accelerate the fault detection and repair process, and achieve automated knowledge graph construction for a shared knowledge base, this study proposes a joint extraction model for track circuit entities and relations, integrating Global Pointer and tensor learning. The model achieved *F*1 scores of 92.1%, 92.7%, and 78.2% on the public datasets NYT, WebNLG, and DuIE, respectively, and was further validated on a private track circuit dataset, demonstrating its potential in real-world maintenance scenarios. The innovations and contributions of this study include the following:

(1) Unlike existing models that only use top-layer outputs from pre-trained models, this research uses a multi-layer dilate gated convolutional neural network (MDGCNN) to extract features from the 12-layer RoBERTa-wwm encoder output. These 12 different levels of semantic information are then adaptively weighted and fused, enhancing the model’s feature representation and improving the recognition accuracy of complex entities and relations.
(2) Existing methods often neglect the deep correlation between multiple relations, especially when multiple entities overlap. To address this, this study applies Tucker decomposition to learn and reconstruct core tensors, subject and object factor matrices, and relation weight matrices, resulting in high-dimensional relation tensors. This not only improves the accuracy of extracting overlapping relations but also strengthens the semantic correlation modeling between different relation types.
(3) The model adopts the Efficient Global Pointer and introduces rotary position encoding (ROPE), along with a multiplicative attention mechanism, to accurately compute entity start and end positions. By sharing weights, the model reduces the number of parameters while maintaining a high recognition performance and reducing time complexity.

The rest of this paper is structured as follows: Section 2 introduces the foundational techniques of early knowledge extraction and reviews the recent research progress in knowledge extraction within the railway domain. Section 3 provides a detailed explanation of the joint extraction model proposed in this paper. Section 4 presents the experimental setup and result analysis, including the visualization of knowledge extraction results from real-world track circuit case data. Section 5 summarizes the main contributions of this study.

## 2. Materials and Methods

### 2.1. Knowledge Extraction

Early researchers treated entity relation extraction as two independent sub-tasks, named entity recognition (NER) and relation extraction (RE) [9]. Although this pipeline extraction approach is flexible, simple to model, and easy to implement, it suffers from several issues, including entity redundancy, high time complexity, difficulty in extracting long dependency relationships between entities, and error accumulation [10,11]. Later, joint entity and relation extraction methods aimed to extract entities and relations in a unified manner through a single model. This approach can improve extraction accuracy by mining implicit associative features between entities and relations. The development of the joint extraction of entities and relations can be divided into two stages: early models based on feature engineering [12,13,14], and more recent joint extraction models based on deep learning. The feature engineering-based models require a large number of manually extracted feature rules, which are costly and inefficient, and they cannot achieve end-to-end joint extraction, making them unsuitable for vertical domain engineering applications. These models have gradually been replaced by deep learning-based joint extraction methods.

Deep learning-based joint extraction methods can be further divided into three categories: (1) Multi-module, multi-step joint extraction models, where the joint extraction task is divided into multiple different modules, and parameter sharing is used to integrate the submodules, thereby enhancing the interaction between them. Zeng et al. [15] proposed a CopyRE joint extraction model to address overlapping triplets in text, which uses a Seq2Seq learning mechanism and copy mechanism to sequentially extract relations, head entities, and tail entities from the text. However, this model requires multiple rounds of copying for texts with overlapping triplets and only considers entities composed of single tokens, leading to poor entity boundary prediction. To address this, Zeng et al. [16] proposed the CopyMTL model in 2020, which builds on the CopyRE model by incorporating multi-task learning to predict entities composed of multiple tokens. Wei et al. [17] innovatively proposed the CasRel model based on a cascading binary tagging framework, modeling relations as functions mapping head entities to tail entities. However, these methods suffer from redundant relation recognition, significantly increasing time complexity, and the random selection of head entities results in instability in unified extraction. Zheng et al. [18] proposed the PRGC model, which decomposes the joint extraction task into three sub-tasks: relation judgment, entity extraction, and subject–object alignment. This effectively alleviated redundant relation judgment and addressed the generalization limitations of span-based extraction and the inefficiency of subject–object alignment. (2) Multi-module, single-step joint extraction models, which use joint decoding algorithms to output triplets. Wang et al. [19] proposed the TPLinker model to address exposure bias and triplet overlap by using a handshake tagging method. This model, based on the connection mechanism between entities, simultaneously performs entity recognition and relation extraction, showing significant advantages in solving triplet overlap and long-distance dependency issues. However, the model uses a complex decoder, resulting in slower convergence, and the issue of relation redundancy remains unsolved. Sui et al. [20] treated entity and relation extraction as a set prediction problem, using the bidirectional encoder representations from Transformers (BERT) as the encoding layer to generate word embeddings and a non-autoregressive decoder as the set generator to predict all triplets at once. This model efficiently resolves the joint prediction problem using a non-autoregressive parallel encoding mechanism and bilateral matching loss function, achieving promising results on public datasets. (3) Single-module, single-step joint extraction models, where fine-grained triplet classification is performed on the text to directly predict all triplets in the corpus in a single step. Shang et al. [21] proposed an end-to-end joint extraction method inspired by the Novel Tagging model structure, achieving fine-grained triplet classification. However, this model can only extract triplet information from single sentences and faces feature conflict issues between entity recognition and relation extraction, resulting in significant computational overhead during training and inference.

### 2.2. Knowledge Extraction in the Railway Domain

As railway systems become increasingly complex and intelligent, multi-source heterogeneous railway maintenance data continue to accumulate. Ensuring that this knowledge is stored systematically and structurally has become a crucial step in improving railway maintenance and management efficiency. Railway knowledge extraction can significantly enhance intelligent maintenance capabilities by extracting key information from unstructured texts such as historical fault reports, maintenance records, and technical standards, forming structured knowledge bases that provide precise decision support for fault diagnosis and the preventive maintenance of railway equipment.

Li et al. [22] proposed a pipeline knowledge extraction model for fault text data of high-speed railway signaling equipment. This method uses BiLSTM+CRF for entity recognition and employs a Transformer network to extract entity relations. Li et al. [23] utilized the BERT-BiLSTM-CRF algorithm for entity recognition in railway construction and combined dependency syntax analysis with a self-attention mechanism to extract both explicit and implicit causal relationships. This approach effectively handles multi-dimensional risk data in complex railway construction areas, generating knowledge graphs that include risk entities and causal relationships. Lin et al. [24] adopted a reinforcement learning strategy to optimize entity extraction. Based on a small-scale manually annotated fault text dataset, they used a reinforcement learning model for sample selection and combined the BERT and BiLSTM-CRF models for entity extraction. In terms of relation extraction, they designed a template-matching method based on domain-specific knowledge characteristics to extract relationships between entities. Subsequently, Lin et al. [25] proposed a multi-module joint extraction model, the RTOM-KE model. This model first employs a two-stage knowledge annotation strategy to label head entities and the corresponding tail entities for each relation. Combining a BERT-base pre-trained model with a bidirectional long short-term memory network (BiLSTM), the model captures multi-dimensional shared representations from the text and extracts contextual features. The head entity extraction module is implemented using a combination of BiLSTM and conditional random field (CRF), while the tail entity extraction module employs a relation gate mechanism to filter the corresponding tail entities and link them with the head entities, forming the final triplet.

## 3. Method

**Figure 2:**
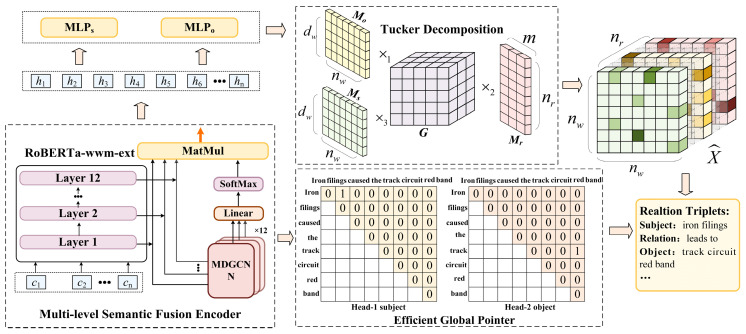
The structure of the joint extraction model for track circuit entities and relations integrates Global Pointer and tensor learning.

The structure of the proposed joint extraction model for track circuit entities and relations, integrating Global Pointer and tensor learning, is shown in Figure 2. First, the model employs a multi-level semantic fusion encoder to perform the upstream encoding task, which encodes the textual information into features. Next, the joint extraction task is treated as a process of Global Pointer search and relation tensor learning. The model uses span-based Efficient Global Pointer to identify the start and end positions of the head and tail entities. The learning and reconstruction of high-dimensional relation tensors are accomplished using the inverse process of Tucker decomposition, which fully captures and learns the relationships between word pairs as well as the semantic associations between different relation types. We summarize frequently used symbols in this article in Table 1.

### 3.1. Multi-Level Semantic Fusion Encoder

The BERT consists of 12 stacked encoder layers with the same structure. According to the results of the probing tasks on the BERT model, it is evident that this model is a hierarchical language learning network, progressing from basic semantic information to higher-level semantic relationships. The lower layers of the network capture surface-level information such as lexical information, the intermediate layers learn syntactic features, and the upper layers analyze advanced semantic information [26]. To fully leverage the multi-level information from pre-trained language models, this paper proposes a multi-level semantic fusion encoder, which extracts the output from each layer of the RoBERTa-wwm-ext pre-trained model [27]. A hidden layer feature extractor is introduced to extract hidden layer features from the 12-layer output of the pre-trained model, and an adaptive mechanism is used to calculate the importance parameters of different semantic information, performing weighted calculations to obtain the final multi-level feature fusion output.

#### 3.1.1. Hidden Layer Feature Extractor

Inspired by the DGCNN network structure [28], this model stacks multiple DGCNN blocks with residual connections. The network introduces spacing between different convolution kernels, allowing it to expand the receptive field and capture information without increasing the number of parameters. Combined with the dynamic adjustment ability of the gating mechanism, the network dynamically selects the convolution outputs, suppressing noisy information and enhancing the model’s ability to capture long-range contextual dependencies. This enables the extraction of features from different levels of semantic information.

**Figure 3:**
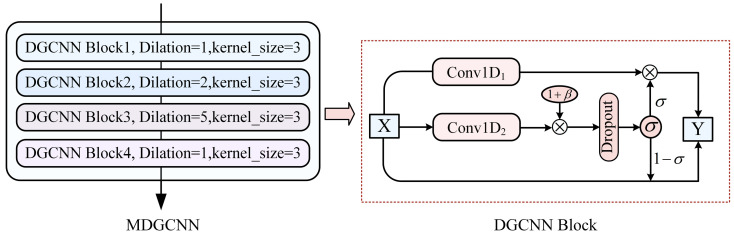
The structure of a multi-layer dilate gated convolutional neural network.

As shown in Figure 3, the multi-layer dilate gated convolutional neural network (MDGCNN) consists of four convolutional layers, each with a kernel size of 3 and dilation rates of (1, 2, 5, 1). Each gated unit in the DGCNN block is composed of two Conv1D layers with identical window sizes but independent weights. Taking DGCNN Block3 as an example, the input sequence X undergoes a dilated convolution with a dilation rate of 5, using a convolution window size of 3. A bias term β [29] is added after the 1D convolution operation, where β follows a normal distribution with a mean of 0 and a standard deviation of 0.1:

(1) $$H=\mathrm{Conv}1D_{2}\left(X\right)⊗\left(1+\beta \right).$$

By applying the dropout function, 10% of neurons are randomly dropped, enhancing the model’s generalization ability. The sigmoid function is used to map the input values into the (0, 1) range, forming a gating mechanism for multi-channel information transmission:

(2) $$Y=X⊗\left(1-\sigma \left({\mathrm{Dropout}}_{p=0.1}\left(H\right)\right)\right)+\mathrm{Conv}1D_{1}\left(X\right)⊗\sigma \left({\mathrm{Dropout}}_{p=0.1}\left(H\right)\right).$$

#### 3.1.2. Multi-Level Semantic Fusion Process

Based on the Transformer architecture, each encoding layer in the pre-trained model captures different semantic and syntactic information. As the semantic layers deepen, how to effectively integrate the outputs of each layer is crucial for improving downstream task performance [30]. The 12-layer outputs of the RoBERTa-wwm-ext pre-trained model are defined as $\left\{H_{1},H_{2},H_{3},\ldots ,H_{12}\right\}$, and the 12 hidden layer representations undergo the multi-layer dilate gated convolutional neural network operations:

(3) $$U_{i}=\mathrm{MDGCNN}\left(H_{i}\right).$$

The weights of $U_{i}$ are dynamically learned through model training, reflecting the relative importance of different layer information in specific tasks. First, a linear transformation is applied to $U_{i}$:

(4) $$\alpha_{i}=\mathrm{ReLU}\left(W^{T}U_{i}+b\right).$$

Next, the softmax function is used to normalize the weight parameters $\alpha_{i}$ into a probability distribution:

(5) $$S_{i}=\mathrm{softmax}\left(\alpha_{i}\right).$$

Then, a weighted fusion is performed to further enhance the richness of the upstream model’s feature representations and its generalization ability, finally obtaining the multi-level semantic fusion output.

(6) $$M=\sum_{i=1}^{12}S_{i}H_{i}.$$

### 3.2. Relation Tensor Learning Module

**Figure 4:**
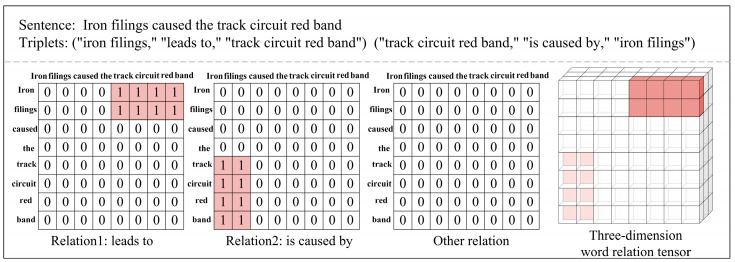
Example of how to construct a three-dimension word relation tensor from word tables.

Many researchers treat relation extraction tasks as table-filling tasks [31,32,33], where the task is represented as a process of filling a 2D matrix, with each cell representing the relationship between two entities (or words). Table-filling provides a structured approach to representing relationships between words, enabling the model to better capture and utilize the global information of the sentence. According to reference [34], the relation extraction task is extended from 2D table-filling to 3D binary tensor learning tasks, as shown in Figure 4. For example, given a set of relationships, the 3D tensor X represents the semantic relationships between word pairs, where $X\in {\mathbb{R}}^{n_{w}\times n_{w}\times n_{r}}$, with $n_{w}$ being the length of the text and $n_{r}$ being the number of relationship types. If the words $w_{u}$ and $w_{v}$ in the text have a relationship $r_{k}$, then $X_{ukv}=1$, otherwise $X_{ukv}=0$. Assuming that the dataset contains a total of three triplets, the relation matrix for this sentence would be filled in two 2D matrices, with the corresponding cells filled with 0 or 1. Finally, all relation matrices are stitched together into a 3D binary tensor X. The goal of model training is to ensure that the model’s predicted output $\hat{X}$ can closely approximate the true tensor X.

Compared to a 2D matrix, a 3D binary tensor can better represent complex relationships within a sentence. A 2D matrix can only learn simple entity pairs and their relations, while a 3D tensor can simultaneously represent multiple types of relationships between multiple entity pairs and capture the diversity of semantics and relations at a higher dimension. By utilizing the basic principles of Tucker decomposition [35], a high-order lexical relation tensor can be decomposed into three low-order factor matrices and a core tensor.

To achieve this, the output from the first stage’s multi-level semantic fusion encoder is encoded in parallel. Two different multilayer perceptrons (MLPs) are used to capture the subject features and object features within the text:

(7) $$\begin{array}{l} M_{s}={\mathrm{MLP}}_{s}\left(H\right),\\ M_{o}={\mathrm{MLP}}_{o}\left(H\right), \end{array}$$

where $M_{s}\in {\mathbb{R}}^{n_{w}\times d_{w}}$ represents the subject feature representation in the text and $M_{o}\in {\mathbb{R}}^{n_{w}\times d_{w}}$ represents the object feature representation in the text.

To obtain a high-dimensional 3D binary relation tensor with rich semantic feature information, we implement it through the inverse process of Tucker decomposition. The lexical relation tensor can be expressed as

(8) $$\hat{X}=\sigma \left(G\times_{1}M_{s}\times_{2}M_{r}\times_{3}M_{o}\right),$$

where σ denotes the sigmoid activation function. $M_{r}\in {\mathbb{R}}^{n_{r}\times d_{r}}$ represents the relation weight matrix, and $G\in {\mathbb{R}}^{d_{w}\times d_{r}\times d_{w}}$ represents the core relation tensor. Both are learnable parameters. By jointly computing G with other factors, the feature information is integrated, enabling the model to learn and capture the semantic relationships between different relation types, thereby predicting and generating the final high-dimensional relation tensor $\hat{X}$.

### 3.3. Efficient Global Pointer Module

#### 3.3.1. Global Pointer

The Global Pointer uses a global unified approach based on cross-boundary regions to recognize nested and non-nested entities. Compared to the CRF model, Global Pointer does not require dynamic programming during inference and avoids recursion, achieving a time complexity of *O*(1) in ideal situations [36].

Assuming a text sequence $C=\left[c_{1},c_{2},\ldots ,c_{n}\right]$ of length n, and utilizing the first-layer multi-level semantic fusion encoder for encoding, we obtain the hidden sequence

(9) $$h_{1},h_{2},\ldots ,h_{n}=\mathrm{MLS}\left(c_{1},c_{2},\ldots ,c_{n}\right).$$

Then, two feedforward neural network encoders are applied for linear transformations to obtain the start and end positions of each entity class, where α represents the entity category, and $q_{i,\alpha }\in {\mathbb{R}}^{d}$, $k_{i,\alpha }\in {\mathbb{R}}^{d}$:

(10) $$\begin{array}{l} q_{i,a}=W_{q,\alpha }h_{i}+b_{q,\alpha },\\ k_{i,\alpha }=W_{k,\alpha }h_{i}+b_{k,\alpha }. \end{array}$$

The score matrix for predicting a span from position i to j in the text for class α is defined as

(11) $$s_{\alpha }\left(i,j\right)=q_{i,\alpha }^{⊤}k_{j,\alpha }.$$

The design of the Global Pointer mainly relies on attention mechanisms to capture the start positions of entities. However, this mechanism does not account for positional information, which may result in sensitivity to the distance between entity boundaries. Without considering the span between two entities, the model might mistakenly predict an entity that spans too far away or might identify one entity’s end position as the start of another [37,38,39]. For example, when two entities are far apart in a text, the model might mistakenly align the end of one entity with the start of a distant entity, generating a “phantom” entity. To alleviate this issue, a new entity encoding is predicted by introducing rotary position encoding (RoPE) [40]. By adding relative position information to the model, this encoding ensures that the model can distinguish boundary positions more precisely. The key update to this encoding mechanism is realized by the following equation:

(12) $$s_{\alpha }\left(i,j\right)={\left(R_{i}q_{i,\alpha }\right)}^{⊤}\left(R_{j}k_{j,\alpha }\right)=q_{i,\alpha }^{⊤}{R_{i}}^{⊤}R_{j}k_{j,\alpha }=q_{i,\alpha }^{⊤}R_{j-i}k_{j,\alpha }.$$

#### 3.3.2. Parameter Reduction

To alleviate the issue of redundant parameters in the Global Pointer model, the Efficient Global Pointer was proposed. It aims to reduce the number of parameters while simultaneously improving the model’s recognition accuracy. By separating the entity extraction task from the classification task, parameter sharing across sub-tasks is achieved, thus reducing the overall number of parameters.

For the entity extraction task, i.e., determining the start and end regions of entities, the same parameters are shared across all entity classes. Therefore, the following equation can be expressed:

(13) $$s_{ext}\left(i,j\right)={\left(W_{q}h_{i}\right)}^{⊤}\left(W_{k}h_{j}\right),$$

where $W_{q}$ and $W_{k}$ are the shared linear transformation matrices, and the parameters remain fixed without varying as the number of entity classes increases.

For the classification task, i.e., determining the entity type, a new linear classifier is introduced. This classifier predicts the entity’s type based on the concatenation result of the start and end representations:

(14) $$s_{\mathrm{cls},\alpha }=w_{\alpha }^{⊤}\left[h_{i};h_{j}\right],$$

where $w_{\alpha }\in {\mathbb{R}}^{2v}$ is the weight vector related to entity type α and $\left[h_{i};h_{j}\right]$ represents the concatenation of the start and end representations.

The final scoring function for the combined extraction and classification tasks is

(15) $$s_{\alpha }\left(i,j\right)={\left(W_{q}h_{i}\right)}^{⊤}\left(W_{k}h_{j}\right)+w_{\alpha }^{⊤}\left[h_{i};h_{j}\right].$$

By concatenating the original input vectors $q_{i,\alpha }$ for the start position and $k_{j,\alpha }$ for the end position, the parameters can be further reduced, and the final scoring function becomes

(16) $$s_{\alpha }\left(i,j\right)=q_{i}^{⊤}k_{j}+w_{\alpha }^{⊤}\left[q_{i};k_{i};q_{j};k_{j}\right].$$

Here, $w_{\alpha }\in {\mathbb{R}}^{4d}$. When a new entity type is added, the number of parameters compared to the original design is reduced to 4d.

### 3.4. Training Strategies

For the relation tensor learning module, there is an issue of class imbalance between positive and negative samples. To address this, the proposed model introduces two strategies to mitigate the effects of unbalanced positive and negative samples. First, a weighting function is introduced to assign different weights to positive and negative samples:

(17) $$\phi \left(x\right)=\left\{\begin{array}{ll} \alpha , & \;\mathrm{if}\;x=0,\\ 1-\alpha , & \;\mathrm{if}\;x=1, \end{array}\right.$$

where x is an element of X and α is a hyperparameter used to control the weighting ratio of positive and negative samples. Positive samples (x=1) are assigned a higher weight of 1−α, while negative samples (x=0) are given a lower weight of α, encouraging the model to focus more on positive samples.

The Focal Loss [41] is employed to further mitigate the impact of class imbalance. Focal Loss is a type of loss function that assigns greater weight to harder-to-predict samples by introducing a tuning factor γ, which adjusts the model’s focus on difficult samples during training. The loss function for the relation tensor learning module is defined as follows:

(18) $$L_{RE}=\sum_{u=1}^{n_{w}}\sum_{k=1}^{n_{r}}\sum_{v=1}^{n_{w}}\phi \left(X_{ukv}\right)\cdot l_{f}\left({\hat{X}}_{ukv}^{P}\right),$$

(19) $$l_{f}\left({\hat{X}}_{ukv}^{P}\right)={\left(1-{\hat{X}}_{ukv}^{P}\right)}^{\gamma }\log\left({\hat{X}}_{ukv}^{P}\right),$$

(20) $${\hat{X}}_{ukv}^{P}=\left\{\begin{array}{ll} {\hat{X}}_{ukv}, & \;\mathrm{if}\;X_{ukv}=1,\\ 1-{\hat{X}}_{ukv}, & \;\mathrm{if}\;X_{ukv}=0, \end{array}\right.$$

where γ is a tuning factor used to control the model’s focus on hard-to-predict samples. As γ increases, the model’s attention to harder samples also increases, reducing the impact of the imbalance caused by the large number of negative samples. ${\hat{X}}_{ukv}$ represents the relationship between the *u*-th and *v*-th word pair under the *k*-th relation type.

(21) $$L_{NER}=\log\left(1+\sum_{\left(i,j\right)\in p_{\alpha }}e^{-s_{\alpha }\left(i,j\right)}\right)+\log\left(1+\sum_{\left(i,j\right)\in Q_{\alpha }}e^{s_{\alpha }\left(i,j\right)}\right),$$

where $P_{\alpha }$ represents the set of starting and ending positions for all entities under the relation type α, and $Q_{\alpha }$ is the set of negative samples. $s_{\alpha }\left(i,j\right)$ represents the predicted score for each entity’s position pair $\left[i:j\right]$.

Finally, in the joint extraction task for both relations and entities using the Global Pointer module, an optimized combined objective function is used during training to simultaneously ensure the accuracy of relation and entity extraction tasks:

(22) $$L=\lambda L_{RE}\left(S\right)+\left(1-\lambda \right)L_{NER}\left(S\right).$$

### 3.5. Inference Strategy

During the inference process, for the entity pair $\left(et^{i},et^{j}\right)$, where $et^{i}=\left\{w_{i_{f}},\ldots ,w_{i_{l}}\right\}$ and $et^{j}=\left\{w_{j_{f}},\ldots ,w_{l_{f}}\right\}$, the Efficient Global Pointer is used to obtain the cross-boundary information $l_{i}$ and $l_{j}$. The relation score between the elements of the entity pair is calculated using the following formula:

(23) $$\frac{1}{l_{i}l_{j}}\cdot \left(\sum_{u=i_{f}}^{i_{l}}\sum_{v=j_{f}}^{j_{l}}{\hat{X}}_{ukv}\right)\geq \delta ,$$

(24) $$\left\{\begin{array}{l} l_{i}=i_{l}-i_{f}+1,\\ l_{j}=j_{l}=j_{f}+1, \end{array}\right.$$

where, if the relation score exceeds the threshold δ, the triplet $\left(et^{i},r_{k},et^{j}\right)$ is considered a highly reliable predicted result.

## 4. Experimentation and Analysis

### 4.1. Experimental Environment and Parameter Configuration

Experimental hardware: The computer operating system is Ubuntu 22.04, with an RTX 4060ti graphics card with 16 GB of VRAM, and an i5-13400 processor. The Python version is 3.10, and the development tool used is PyCharm. This track circuit joint extraction model was built using the Pytorch deep learning framework, and the model network parameters are shown in the Table 2:

### 4.2. Experimental Evaluation Metrics

This experiment uses precision (*P*), recall (*R*), and the *F*1 score as evaluation metrics for the joint extraction model of the track circuit maintenance text. Here, *TP*, *FP*, and *FN* are the numbers of true positives, false positives, and false negatives, respectively.

(25) $$P=\frac{TP}{TP+FP}\times 100\%,$$

(26) $$R=\frac{TP}{TP+FN}\times 100\%,$$

(27) $$F1=\frac{2\cdot P\cdot R}{P+R}\times 100\%.$$

### 4.3. Presentation of Experimental Data

The subject of this study is the track circuit maintenance text data from multiple railway bureau jurisdictions, covering the past five years of track circuit repair process records. These records involve information such as track circuit fault phenomena, fault locations, fault cause analyses, and the repair measures taken in response to the fault phenomena and their causes. Maintenance text data are typically compiled by on-site duty personnel based on their operational experience and fault-handling practices. Due to differences in the technical backgrounds, language expression habits, and approaches to handling faults among the personnel, the format and content of the records are not standardized.

**Figure 5:**
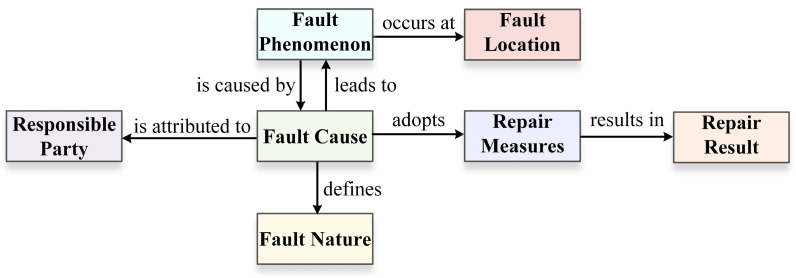
Knowledge association structure diagram.

Therefore, this paper analyzes and statistically processes 530,000 characters of maintenance text corpus, identifying seven entity types and seven relationship types. The knowledge association structure composed of entities and their relationships is shown in Figure 5, and the track circuit entity–relation matching template is presented in Table 3.

### 4.4. Comparison Experiment of Joint Entity–Relation Extraction Using Public Datasets

To verify the generalization capability of our model, we conducted tests on the English public datasets NYT [42] and WebNLG [43], as well as the Chinese public dataset DuIE [44]. The NYT dataset is derived from *The New York Times* and contains 24 types of relations, while the WebNLG dataset includes 246 types of relations and was initially created for the Natural Language Generation (NLG) task. The DuIE dataset, released by Baidu, is a large-scale manually annotated dataset containing 48 types of relations.

To further validate the effectiveness of our model in extracting relational triplets, we compared it with different baseline models, as follows:

(1) CopyRe [15]: This model adopts a seq2seq architecture and incorporates a copy mechanism to address the issue of extracting long-tail relationships;
(2) MultiRe [45]: This model combines a seq2seq framework with reinforcement learning to extract relational triplets;
(3) CopyMTL [16]: This model is a further improvement on CopyRe, where the decoder uses an attention-fused LSTM model, and a fully connected layer is employed to obtain the output;
(4) GraphRel [46]: This model uses a relation-weighted graph convolutional neural network (GCN) to model the interactions between named entities and their relationships;
(5) CasRel [17]: This model is a sequence labeling model. It extracts relational triplets through a cascading framework. First, it predicts the subject, and then, based on the predicted subject, it predicts the related relations and objects;
(6) TPLinker [19]: This model introduces a handshake tagging paradigm, which cleverly divides token pair links into three types, effectively addressing the issue of exposure bias in relation extraction;
(7) TLRel [34]: This model uses BiLSTM to encode the input text. In the entity recognition module, CRF is used to output valid sequence tags, while in the relation extraction module, the model innovatively employs Tucker decomposition for tensor learning.

Based on the experimental results in Table 4, it can be seen that the proposed model demonstrates strong generalization capabilities, achieving *F*1 scores of 92.1% on the NYT dataset, 92.7% on the WebNLG English public dataset, and 78.2% on the DuIE Chinese public dataset. The performance using the BERT model as the upstream encoder is superior to that of using an LSTM model, further emphasizing the importance of selecting the right upstream encoder to enhance the overall performance of the model. The results indicate that the proposed model, with its multi-level semantic fusion encoder, exhibits powerful feature extraction capabilities, allowing it to better adapt to different domains and datasets with complex structures.

### 4.5. Experimental Analysis Based on Track Circuit Dataset

**Figure 6:**
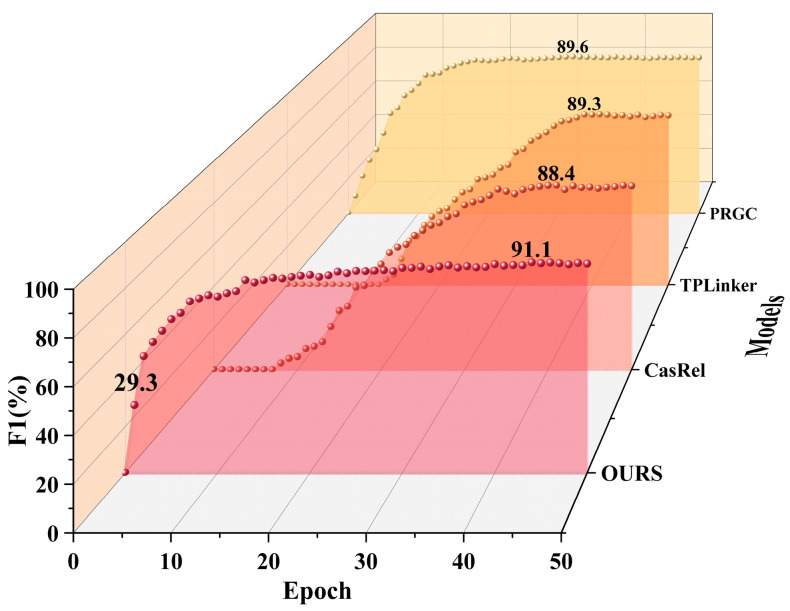
The results of different methods on the track circuit validation set.

The track circuit maintenance text data exhibit characteristics such as diversity, subjectivity, lack of standardization, and varying data quality, leading to significant noise in the dataset. This increases the difficulty of information extraction and processing. On the track circuit dataset, we trained the proposed model for 50 epochs and compared it with the CasRel, TPLinker, and PRGC models. The comparison results are shown in Figure 6.

From Figure 6, it can be concluded that the proposed model converges quickly, achieving an *F*1 score of 29.3% for triplet extraction at Epoch 1 (i.e., after a complete pass through the entire dataset), with stable and efficient performance growth. The CasRel and TPLinker models have larger parameter sizes and a higher complexity, which results in a lower training efficiency and slower performance improvements. These models require more training epochs to approach the performance of the proposed model. Although the PRGC model converges relatively quickly in the early stages, its overall performance is limited. In contrast, the proposed model significantly reduces training time and resource requirements under the same conditions, showing superior stability and effectiveness. Ultimately, the proposed model achieves optimal performance at Epoch 44, with an *F*1 score of 91.1% on the validation set.

#### 4.5.1. Experimental Analysis of the Upstream Encoding Module

To further validate the effectiveness of the proposed multi-level semantic fusion encoder, we represent this encoder as RoBERTa+HIRE (MDGCNN). Comparative experiments were conducted by replacing different upstream encoders.

**Figure 7:**
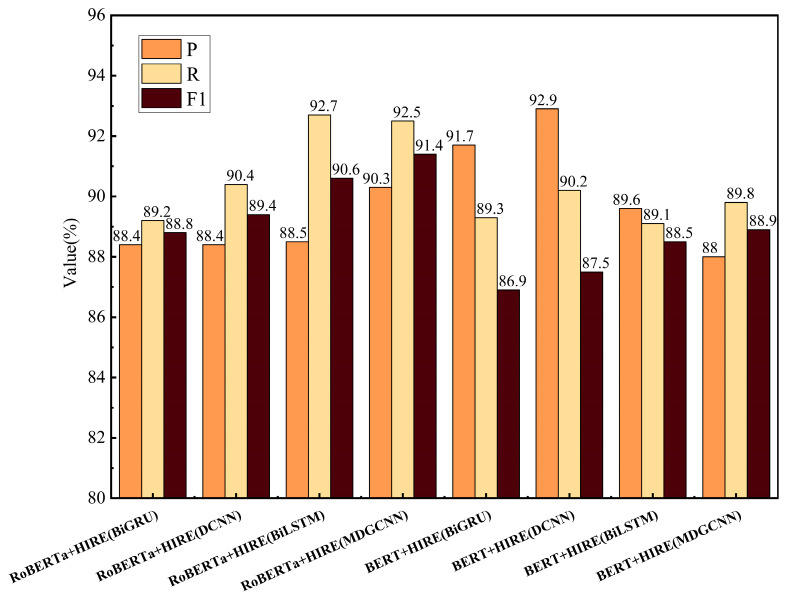
The experimental results using different upstream models on the track circuit test set.

The basic encoders used for comparison are RoBERTa-wwm-ext and BERT, and the hidden layer feature extractors used are BiGRU (bidirectional gated recurrent unit), BiLSTM, and DCNN (dilated convolution) [47], with a dilation rate of (1,2,5) and a convolution kernel size of 3. From the results shown in Figure 7, it can be further concluded that using MDGCNN as the hidden layer feature extractor, compared to BiGRU, BiLSTM, and DCNN, can extract semantic features at different scales, enhancing the contextual perception range. The gating mechanism effectively suppresses noise information in the text, enhancing the information extraction capabilities. Although BiGRU and BiLSTM have some ability to capture context, their receptive fields are limited and cannot effectively expand the feature capture range. Moreover, BiGRU and BiLSTM have higher computational complexity, whereas MDGCNN, through dilated convolutions, reduces the time complexity and is more advantageous in capturing long-distance dependencies.

To validate the impact of the dilation rate of the MDGCNN network in the multi-level semantic fusion encoder on model performance, we set the dilation rates d of the MDGCNN network to 1,1,2,1; 1,2,4,1; and 1,2,5,1. The experimental results are shown in Table 5.

Based on the results of Table 5, it can be seen that the model performs best when the dilation rate of the MDGCNN network in the multi-level semantic fusion encoder is set to (1,2,5,1). In this configuration, the first layer of the MDGCNN is a standard convolution, primarily used to capture local semantic details. The dilation rate of the second layer is set to 2, and the dilation rate of the third layer is set to 5, further expanding the receptive field, allowing the model to extract a broader range of contextual information without significantly increasing the computational burden. The *F*1 score of MDGCNN(1,2,5,1) is 0.03% higher than that of MDGCNN(1,2,5), indicating that restoring the dilation rate to 1 in the fourth layer allows for the fine-tuning of fine-grained details. This adjustment enables the integration of local and global information, ensuring that detailed features are not lost.

#### 4.5.2. Experimental Analysis of the Relation Tensor Learning Module

**Figure 8:**
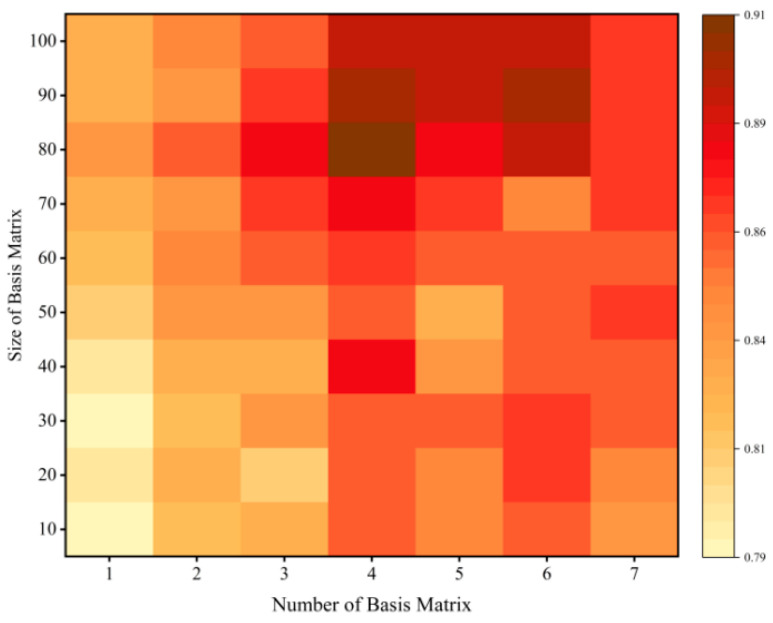
The triplet extraction performance under different dimensions of the core tensor G.

In the relation tensor learning module, the core relation tensor $G\in {\mathbb{R}}^{d_{w}\times m\times d_{w}}$ contains m base matrices. Each base matrix is $d_{w}\times d_{w}$ in size. The base matrices are mainly used to describe the association features between different types of relations. To evaluate the impact of the number of base matrices m and the dimensionality $d_{w}$ on model performance, tuning experiments were conducted, and the changes in the model’s *F*1 score are shown using the heatmap in Figure 8.

As can be seen from Figure 8, the model performs well in the upper half of the heatmap when m=4 and the dimensionality $d_{w}=80$. Reducing either the number of base matrices m or the dimensionality $d_{w}$ results in insufficient parameters for the tensor G, preventing the model from effectively capturing the correlations between relations in the text. Conversely, increasing these parameters too much can lead to overfitting and weaken the model’s generalization ability.

In the tensor learning module, the distribution of positive and negative samples is highly imbalanced, making the model more prone to overfitting the majority class. To address the imbalance in sample distribution, the learning rate for the current model was fixed, and tuning experiments were conducted on the hyperparameters α and γ.

**Figure 9:**
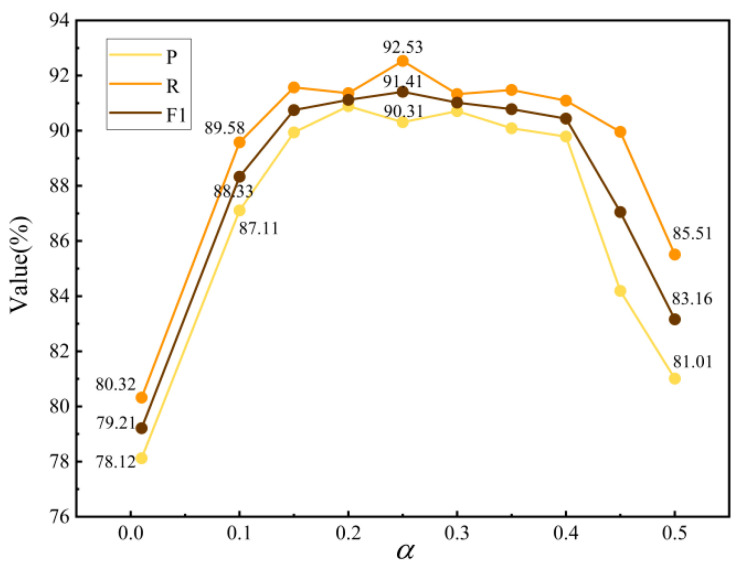
The parameter-tuning experiment for α.

The candidate values for hyperparameter α were selected from the following set: {0.01, 0.10, 0.15, 0.2, 0.25, 0.3, 0.35, 0.4, 0.45, 0.5}. The tuning results are shown in Figure 9. When α=0.01, the positive sample weighting is too high, leading to a higher metric *P* but lower metric *R* and *F*1 score. This suggests that the model is biased towards recognizing positive samples but is less effective at handling negative samples, leading to a reduced overall precision.

When α is increased to between 0.1 and 0.2, the values of *P*, *R*, and *F*1 reach a balanced state, with the highest scores observed around α=0.25, indicating that this value provides the optimal balance between positive and negative samples. The model is then able to handle the imbalance in the sample distribution more effectively, resulting in the best classification performance. Further increasing α beyond 0.25 increases the model’s focus on negative samples, leading to a decline in overall extraction performance.

**Figure 10:**
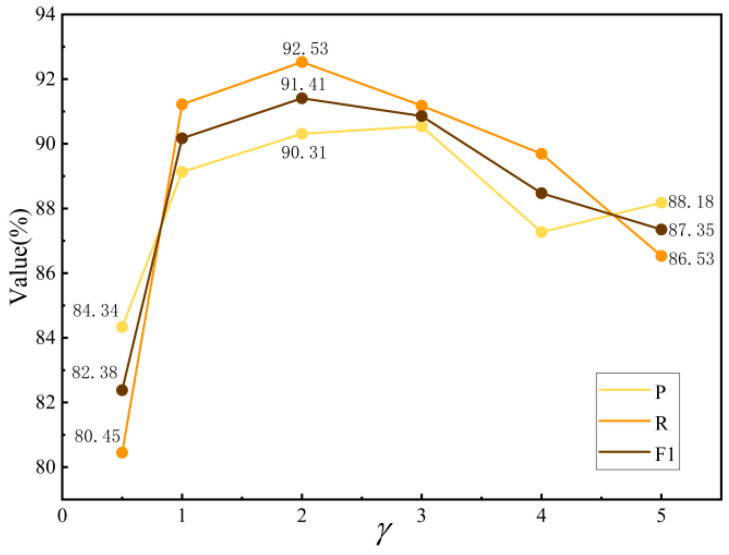
The parameter-tuning experiment for γ.

Based on the results of the tuning experiment shown in Figure 10, when γ is less than 1, the three evaluation metrics are relatively low, indicating that the model handles easy-to-classify and difficult-to-classify samples in a relatively balanced manner. When γ=2, the model’s overall classification performance reaches its optimal state. However, if the γ value continues to increase, the model overly focuses on difficult-to-classify samples, causing the overall performance to decline.

#### 4.5.3. Experimental Analysis of the Efficient Global Pointer Module

Unlike traditional absolute position encoding, RoPE encoding effectively introduces relative positional information within the self-attention mechanism, allowing the model to better capture the relative relationships between elements in a sequence. Additionally, RoPE achieves a high degree of flexibility in sequence length through its rotational operation, making the model’s generalization ability stronger when handling long sequences without being constrained by length.

CRF, as a commonly used discriminative probabilistic model for sequence labeling tasks, is particularly well suited for handling dependencies in sequence data. As shown in the experimental results in Table 6, the metric *P* reached 90.99% in the experiment. However, due to its limited ability to handle long sequences and complex dependencies, the overall performance was relatively low. Compared to the CRF model, Global Pointer combined with RoPE encoding showed a significant improvement in both precision and *F*1 score, with the *F*1 score reaching 90.92%. The RoPE encoding introduces relative positional information through rotational operations, giving the model greater flexibility when handling long sequences and improving its ability to capture complex entities. In comparison to Global Pointer, the *F*1 score of the Efficient Global Pointer model increased from 90.92% to 91.28%, further demonstrating the effectiveness of the Efficient Global Pointer in capturing global information. The Efficient Global Pointer model, combined with RoPE positional encoding, enhances the model’s robustness in handling recognition tasks with complex structures by capturing relative positional information and optimizing computational complexity.

### 4.6. Case Study

**Figure 11:**
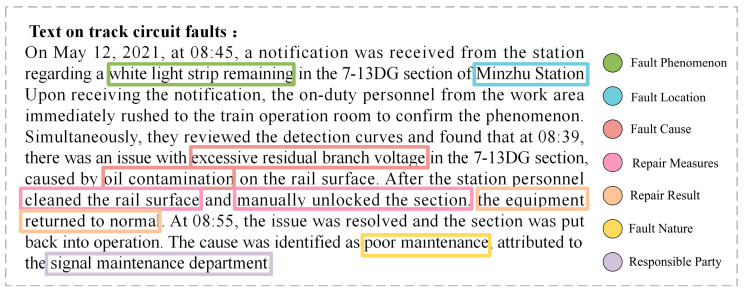
The model extracts entity types from case sentences.

**Figure 12:**
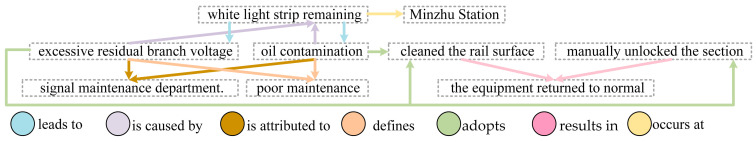
The model extracts relation types from case sentences.

To validate the practical applicability of the model proposed in this paper, and to specifically and intuitively reflect its ability in overlapping triplet extraction, as well as to provide important node data support for the subsequent construction of a knowledge graph for railway signal equipment faults, case sentences were selected from the track circuit dataset, and triplets were extracted. The extracted entities and relations are visualized in Figure 11 and Figure 12, respectively.

## 5. Conclusions

In this paper, we propose a joint extraction model that integrates Global Pointer and tensor learning to address the challenges of extracting entities and relations from track circuit maintenance text data. The model uses a multi-level semantic fusion encoder to extract semantic features at different levels and combines Tucker decomposition and Global Pointer techniques to accurately extract complex triplet information. Based on the experimental results, the following conclusions were drawn:

(1) The multi-level semantic fusion encoder integrates a whole-word masking strategy and a multi-level semantic fusion approach. Compared to baseline models, this model better understands complex semantic structures in the text, enhances the ability to capture contextual information, and effectively suppresses noise interference in the text, thereby improving the overall performance of the model.
(2) The tensor learning module employs the Tucker decomposition method to effectively capture the semantic associations between different types of relations. Compared to traditional relation extraction methods, the tensor learning module uses a three-dimensional tensor representation to better handle the complex relationships between multiple entity pairs within a sentence. Additionally, this module significantly reduces the computational complexity, improving training efficiency on large datasets.
(3) The Global Pointer module efficiently identifies entities in the text through a span-based global normalization mechanism. Unlike traditional sequence labeling models (such as CRF), this module does not require recursive denominator computation, greatly improving its computational efficiency. Furthermore, the Efficient Global Pointer separates the entity extraction task from the classification task, allowing parameter sharing. By introducing RoPE encoding, the computational burden is further reduced while maintaining recognition accuracy.

In summary, the model proposed in this paper demonstrates significant adaptability and robustness when addressing issues such as nested entities, overlapping relationships, and data imbalance, while effectively reducing training time and complexity. The experimental results on the track circuit dataset indicate that the model outperforms existing mainstream methods, achieving higher *F*1 scores. This provides reliable technical support for constructing knowledge graphs in the railway maintenance domain and has important practical application value.

## Figures and Tables

**Table 1:** Definitions of symbols used in this paper.

Symbol	Definition
X	Gold binary word relation tensor
$\hat{X}$	Predicted binary word relation tensor
G	Core relation tensor
$M_{s}$/$M_{o}$	Subject–object entity feature matrices
$M_{r}$	Relational weight matrix
$d_{w}$	Word feature dimension
$n_{w}$	The number of words
$n_{r}$	The number of relation
σ	The activation function
$\times_{n}$	Tensor *n*-mode product
$h_{n}$	Feature vector of upstream output
Conv1D_1_/Conv1D_2_	1D convolution
β	A bias term
$q_{i,\alpha }$	The start positions of each entity class
$k_{i,\alpha }$	The end positions of each entity class
$s_{\alpha }\left(i,j\right)$	The score matrix

**Table 2:** Network parameters of the track circuit joint extraction model.

Parameter Name	Parameter Value
DilatedGatedConv1d_dim	128
MDGCNN convolution kernel size	3
MDGCNN dilation rate	(1,2,5,1)
Dropout	0.1
Upstream model learning rate	2 × 10^−5^
Downstream model learning rate	1 × 10^−4^
Efficient_Global_pointer_inner_dim	64
MLPs_dim	256
MLPo_dim	256
δ	0.9
λ	0.5
Batch_size	24
Epoch	50
Max_seq_len	256

**Table 3:** Track circuit entity–relation matching template (partial).

Entity A	Relation	Entity B	Example of Extracted Triplet Result
Fault Phenomenon	occurs at	Fault Location	<Track circuit red band, occurs at, Fuqiang Station>
Fault Phenomenon	is caused by	Fault Cause	<Residual green band, is caused by, vehicle wheel slippage>
Fault Cause	leads to	Fault Phenomenon	<Breaker failure, leads to, track circuit red band>
Fault Cause	is attributed to	Responsible Party	<Rail Break, attributed to, Workers>
Fault Cause	adopts	Repair Measures	<Wear plate defect, adopts, wear plate replacement>
Fault Cause	defines	Fault Nature	<Capacitor failure, defines, poor inspection>
Repair Measures	results in	Repair Result	<Enabled axle counting device, results in, submitted for use>

**Table 4:** Performance comparison of different methods on public datasets NYT, WebNLG, and DuIE.

Models	NYT Dataset			WebNLG Dataset			DuIE Dataset		
	*P* (%)	*R* (%)	*F*1 (%)	*P* (%)	*R* (%)	*F*1 (%)	*P* (%)	*R* (%)	*F*1 (%)
CopyRe	61.0	56.6	58.7	37.7	36.4	37.1	39.7	38.9	39.3
MultiRe	77.9	67.2	72.1	63.3	59.9	61.6	49.1	47.7	48.4
CopyMTL	75.7	68.7	72.0	58.0	54.9	56.4	44.8	43.2	44.0
GraphRel	63.9	60.0	61.9	44.7	41.1	42.9	37.8	39.6	38.7
CasRel_BERT_	89.7	89.5	89.6	**93.4**	90.1	91.8	72.3	74.3	73.3
TPLinker_BERT_	**91.3**	92.5	91.9	91.8	92.0	91.9	73.4	75.2	74.3
TLRel	88.5	85.2	86.8	91.8	**92.7**	92.2	76.1	78.7	77.4
**Ours**	90.93	**93.3**	**92.1**	92.9	92.5	**92.7**	**77.5**	**78.9**	**78.2**

**Table 5:** MDGCNN parameter-tuning experiments.

Parameter Settings	*P* (%)	*R* (%)	*F*1 (%)
MDGCNN(1,1,2,1)	90.78	91.89	91.33
MDGCNN(1,2,4,1)	90.27	91.52	90.89
**MDGCNN(1,2,5,1)**	90.31	**92.53**	**91.41**
MDGCNN(1,1,2)	90.80	91.74	91.27
MDGCNN(1,2,4)	89.71	91.63	90.66
MDGCNN(1,2,5)	**91.08**	91.68	91.38

**Table 6:** Comparison experiments of the Efficient Global Pointer module.

Models	*P* (%)	*R* (%)	*F*1 (%)
CRF	90.99	88.56	89.76
Global Pointer (+ROPE)	90.36	91.50	90.92
Efficient Global Pointer	**90.81**	91.75	91.28
**Efficient Global Pointer (+ROPE)**	90.31	**92.53**	**91.41**

## Data Availability

The data presented in this study are available on request from the corresponding author.
